# An image of a giant brown tumor due to severe secondary hyperparathyroidism

**DOI:** 10.1590/2175-8239-JBN-2024-0083en

**Published:** 2024-09-06

**Authors:** Ana Carolina de Lima, Ana Claudia Siqueira Marques, Rodrigo Bueno de Oliveira

**Affiliations:** 1 Universidade Estadual de Campinas, Faculdade de Ciências Médicas, Departamento de Medicina Interna, Divisão de Nefrologia, Campinas, SP, Brazil.; 2 Universidade Estadual de Campinas, Faculdade de Ciências Médicas, Laboratório para o Estudo Mineral e Ósseo em Nefrologia, Campinas, SP, Brazil.

**Figure 1:**
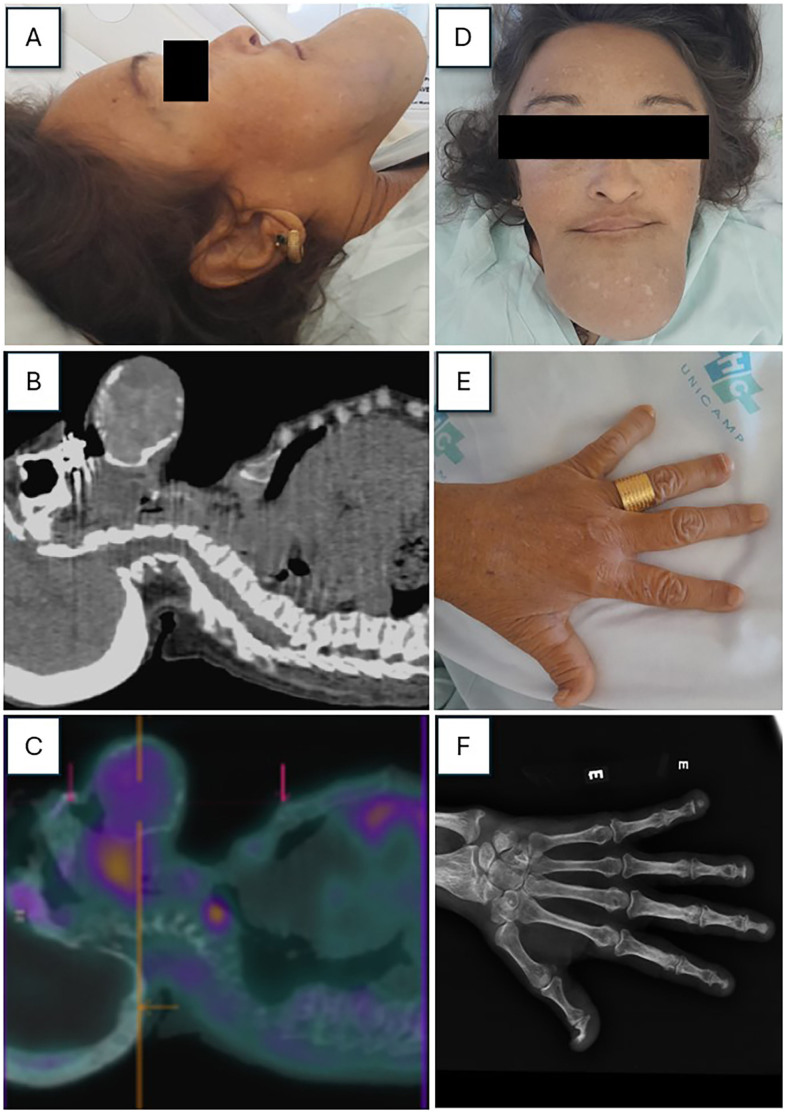
A and D, Giant tumor in the mandibular region, involving the lower dental arch with loss of teeth and bulging and protrusion of the palate; B and C, The 2-hour ^99m^TC-MIBI Spect/CT images show areas of hyper uptake of the radiopharmaceutical in nodular formation located between the esophagus and posterior trachea at C7 level measuring 1.8 cm, compatible with ectopic parathyroid hyperplasia, and expansive heterogeneous bone lesions (brown tumors) in the maxillary sinus, mandibular and mental regions, with disruption of the cortex and involvement of soft tissues in the mandible; E and F, Deformities of the distal phalanges, shortening due to bone resorption, deviations, diffuse bone demineralization, and deformities suggesting osteitis fibrosa.

A 49-year-old woman with chronic kidney disease on hemodialysis since 2004 sought medical attention due to bone pain, fractures (pelvis, femur), and deformities in her hands and face. Two years after subtotal parathyroidectomy, she reported that the mandible lesion had progressed (Figure 1). Laboratory test results were: serum parathormone 1227 pg/mL (15–65 pg/mL), phosphate 4.5 mg/dL (2.5–4.5 mg/dL), total-calcium 8.2 mg/dL (8.8–10.6 mg/dL), and alkaline-phosphatase 817 IU/L (33–98 IU/L). ^99m^TC-MIBI Spect/CT-scintigraphy^1,2,3^ revealed a nodular image in the tracheoesophageal sulcus (ectopic parathyroid adenoma) and expansive bone lesions (“brown tumors”), with cortex disruption and soft tissue involvement in the mandible.
