# Supplementary material for: An image of a giant brown tumor due to severe secondary hyperparathyroidism
Source: J Bras Nefrol. 2024 Sep 6;46(3):e20240083. doi: 10.1590/2175-8239-JBN-2024-0083en (PMC11398717; doi:10.1590/2175-8239-JBN-2024-0083en)
Supplement: Translation

# Translation: Imagem de tumor marrom gigante devido a hiperparatireoidismo secundário grave

**DOI:** 10.1590/2175-8239-JBN-2024-0083pt

**Authors:** Ana Carolina de Lima, Ana Claudia Siqueira Marques, Rodrigo Bueno de Oliveira

**Affiliations:** 1 Universidade Estadual de Campinas, Faculdade de Ciências Médicas, Departamento de Medicina Interna, Divisão de Nefrologia, Campinas, SP, Brasil.; 2 Universidade Estadual de Campinas, Faculdade de Ciências Médicas, Laboratório para o Estudo Mineral e Ósseo em Nefrologia, Campinas, SP, Brasil.

Uma mulher de 49 anos com doença renal crônica, em hemodiálise desde 2004, procurou atendimento médico devido a dores ósseas, fraturas (pelve, fêmur) e deformidades nas mãos e no rosto. Dois anos após a paratireoidectomia subtotal, ela relatou que a lesão na mandíbula havia progredido (Figura 1). Os resultados dos exames laboratoriais foram: paratormônio sérico 1227 pg/mL (15–65 pg/mL), fosfato 4,5 mg/dL (2,5–4,5 mg/dL), cálcio total 8,2 mg/dL (8,8–10,6 mg/dL) e fosfatase alcalina 817 UI/L (33–98 UI/L). A cintilografia com ^99m^TC-MIBI Spect/CT^1–3^ revelou imagem nodular no sulco traqueoesofágico (adenoma de paratireoide ectópico) e lesões ósseas expansivas (“tumores marrons”), com destruição cortical e envolvimento de tecidos moles na mandíbula.

**Figura 1:** A e D: Tumor gigante na região mandibular, envolvendo o arco dentário inferior com perda de dentes e abaulamento e protrusão do palato; B e C: As imagens de 2 horas do ^99m^TC-MIBI Spect/CT mostram áreas de hipercaptação do radiofármaco em formação nodular localizada entre o esôfago e a traqueia posterior no nível C7, medindo 1,8 cm, compatível com hiperplasia paratireoideana ectópica, e lesões ósseas expansivas heterogêneas (tumores marrons) no seio maxilar, nas regiões mandibular e mental, com destruição cortical e envolvimento de tecidos moles na mandíbula.; E e F: Deformidades das falanges distais, encurtamento decorrente de reabsorção óssea, desvios, desmineralização óssea difusa e deformidades sugestivas de osteíte fibrosa.
